# Assessment of Thermal Performance of Textile Materials Modified with PCM Microcapsules Using Combination of DSC and Infrared Thermography Methods

**DOI:** 10.3390/molecules25010122

**Published:** 2019-12-28

**Authors:** Alicja Nejman, Ewa Gromadzińska, Irena Kamińska, Małgorzata Cieślak

**Affiliations:** ŁUKASIEWICZ Research Network—Textile Research Institute, Scientific Department of Unconventional Technologies and Textiles, 5/15 Brzezinska St., 92-103 Lodz, Poland; egromadzinska@iw.lodz.pl (E.G.); ikaminska@iw.lodz.pl (I.K.); cieslakm@iw.lodz.pl (M.C.)

**Keywords:** textile materials, phase change materials, microcapsules, differential scanning calorimetry, infrared thermography

## Abstract

The aim of the study was the combination of two measurement methods, the differential scanning calorimetry (DSC) and infrared thermography to evaluate thermal performance of woven and knitted fabrics coated with acrylic pastes containing 20% (P/20) and 40% (P/40) of microcapsules of phase change materials (MPCM) with transition temperatures of 28 °C (MPCM28) and 43 °C (MPCM43). The DSC analysis showed that the phase transition processes for materials modified with pastes P/20 occur in a narrower temperature range than those modified with P/40 pastes. The initial temperatures *T_Onset (S-S)_* and *T_Onset (S-L)_* are higher for materials modified respectively with pastes P/20 and P/40. The melting and crystallization enthalpy values of both P/20 coated materials are lower by about 45% and 35% compared to P/40. Infrared thermography analysis showed that materials modified with P/20 are heating up faster than modified with P/40 for both MPCM. In the cooling process for modified fabrics the highest temperature decrease was observed in the first 30s. Materials modified with paste P/40 were cooled more slowly in comparison with paste P/20, both for MPCM28 and MPCM43.

## 1. Introduction

One of the possible ways of producing functional textile materials is the incorporation of the modifiers in the microcapsules form [1,2,3,4,5,6,7,8,9,10,11] into the material structure, in which many active substances can be entrapped. One of the groups of such substances are phase change materials (PCM), which can shape thermoregulatory performance and improve thermal comfort. They have the ability to absorb and store large amounts of heat during heating and its releasing during cooling. Phase change materials may be organic compounds (alkanes) or inorganic compounds (e.g., hydrated inorganic salts) [12,13]. About 500 potential phase change materials are known [5]. Alkanes with a carbon number from 16 to 20, such as *n*-Hexadecane (T_m_ = 18.2 °C), *n*-Heptadecane (T_m_ = 22.5 °C), *n*-Octadecane (T_m_ = 28.2 °C), *n*-Nonadecane (T_m_ = 32.1 °C) or *n*-Eicosan (T_m_ = 36.1 °C) are the most often used. Used PCM are entrapped in microcapsules with a diameter of few micrometers [14,15]. Textile material modified with PCM can absorb or emit heat under the changing of ambient conditions influence, in different temperature ranges which depend on the phase change temperature of the used PCM. Several technologies for PCM microcapsules incorporation into the textile structure are known, such as padding, printing, coating. The most common is microcapsules dispersing in binding agents, then applying such dispersion using e.g., padding, coating, printing, spraying techniques [7].

The differential scanning calorimetry (DSC) technique is the most often used for the thermal performance of such textiles. Thermal effects for textiles modified with homogeneous PCM microcapsules and their blends with different phase transition temperatures, influence of the PCM microcapsules incorporation technique (coating, printing and surfacing) into textile structure and the influence of heating/cooling speed on thermal properties assessment of MPCM modified materials were investigated [6,7,8]. For the assessment of thermal performance characteristic of textiles with PCMs are used both experimental investigation and mathematical modeling [9].

The infrared thermography technique is also used for thermal effects study. In this method changes in the temperature difference between the test and reference samples are measured. Using this technique the temperature changes on the surface of textile materials with a polymer coating with and without PCM microcapsules were examined, respectively. The researches were conducted for textile materials made of polyamide, cotton and mixtures of various fibers [16,17,18,19,20].

The aim of the work was to combine two measurement methods, the DSC analysis and infrared thermography technique to evaluate thermal performance of two different textile structures (woven and knitted fabrics) modified under the same conditions with two different PCM microcapsules with phase transition temperature of 28 °C (MPCM28) and 43 °C (MPCM43). The differential scanning calorimetry (DSC) studies allow to obtain the basic thermal properties of MPCM modified textile materials. The use of infrared thermography technique allows the assessment of the thermal properties in real conditions. The combination of both techniques is important to assess their full thermal characteristics.

## 2. Results and Discussion

### 2.1. SEM Analysis

**Figure 1:**
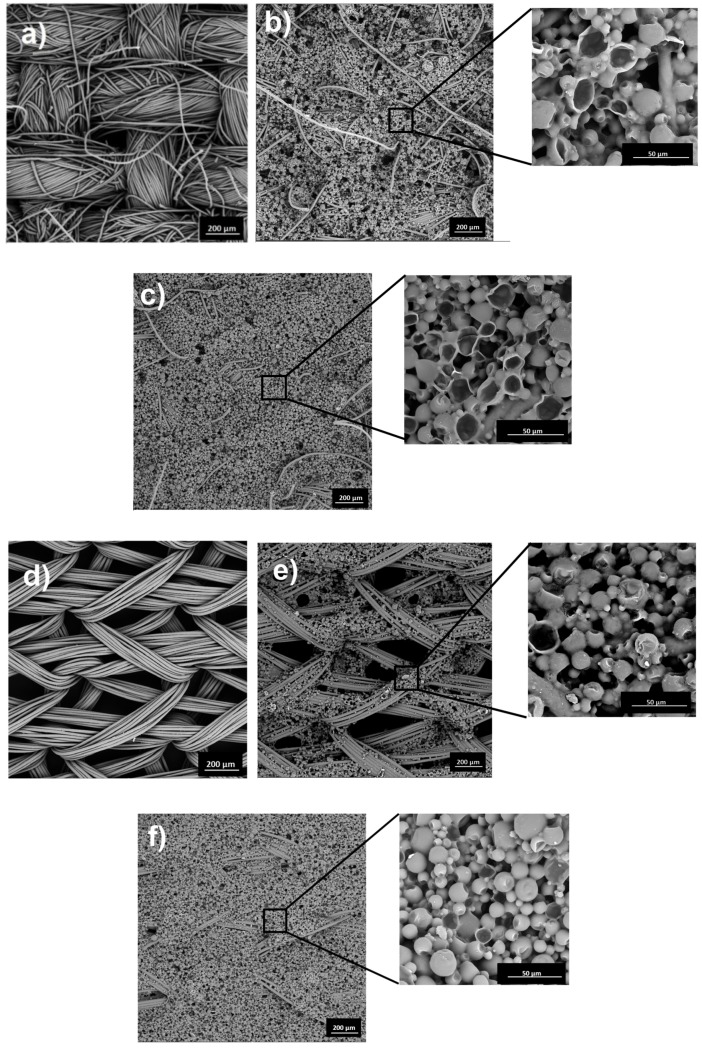
SEM images of the surface of: (**a**) raw and modified woven fabric (W): (**b**) W28/20, (**c**) W28/40 and (**d**) raw and modified knitted fabric (K): (**e**) K28/20, (**f**) K28/40 (magnification 150×, 1000×).

The raw woven fabric (Figure 1a) has a denser structure than the raw knitted fabric (Figure 1d), which has larger air spaces. The surface of modified W fabrics is covered evenly. Due to the denser fabric structure, the coating layer is mainly on the surface, not entering the spaces between the weft-warp and interfibrillar systems. The W fabric with paste P/20 (Figure 1b) is covered with a thin layer of MPCM paste as evidenced by many fibers protruding above the modified surface. The coating layer of W fabric with paste P/40 is thicker (Figure 1c), we observe single fibers above the coating surface. In turn, the surface of K fabric is coated unevenly due to significant air spaces in the structure. During the coating process, paste with MPCM is incorporated into the fibers in air spaces and on the fabric surface, which causes unevenness. On the Figure 1f we observe a greater coating of the K fabric structure with paste P/40 than with paste P/20 (Figure 1e), for which MPCM are observed in the fibers inside the air spaces. For all modified fabrics, the presence of damaged MPCMs is observed. Some of the microcapsules on the surface are damaged by the action of the coating knife. This represents a small and comparable percentage for all samples. Inside the coating layer, the microcapsules were not damaged. MPCM used for fabrics modification have regular, spherical shapes [11]. Only damaged microcapsules on the coating layer surface are cracked, what is an effect of coating process. Damages of microcapsules during the pastes application on dense woven fabric structure are greater than to the more porous knitted fabric structure, as seen in Figure 1b,c,e,f. Plain material W has an ordered, compact structure and relatively less airspace than material K, which results in greater interaction with the paste during coating. MPCMs on the surface are more susceptible to damage than on K materials, where microcapsules can also enter the spaces between the fibers, which protect them from damage (Figure 1a,d). The resulting defects were not observed in our previous studies [6,7,8], in which printing, padding and coating techniques using a 0.1 mm gap were applied [7]. In this research the smaller gap (0.05 mm) cause more pressure on microcapsules, which is why they are partially damaged, especially on the dense woven fabric.

### 2.2. DSC Analysis

Results of DSC investigations showed that melting and crystallizing processes of powders take place in wider temperature ranges than for modified W and K fabrics (Table 1). The initial temperature of the solid-solid transition (*T_Onset (S-S_*_)_) of MPCM28 for melting is lower and for crystallization higher than for the modified materials. In the case of MPCM43 powder, the temperature *T_Onset (S-S)_* is comparable, and for crystallization higher than MPCM on the materials. The initial temperatures of the solid-liquid transition (*T_Onset (S-L)_*) and the initial crystallization temperatures of both MPCM powders are higher, and the final temperatures are lower than for MPCM modified materials. The temperatures for powders at peaks maxima are higher for melting and lower for crystallization in comparison with MPCM modified materials. This is due to the fact that the amount of MPCM powder is the highest and in the case of powders, heat is delivered directly to the microcapsules. Therefore, the temperature *T_Onset (S-S_*_)_ is lower than for the modified textile materials. In the case of W and K fabrics, acrylic layer and the structure of the textile material have influence on the heat transport to MPCM and the temperature values. These may likely result because of differences in the thermal conductivity of individual components of the tested material and their share. The MPCM powder consist a melamine-formaldehyde shell and PCM paraffin filling. The thermal conductivity of melamine-formaldehyde resin is 0.2 W/mK, while *n*-Octadecane and n-Docosane is 0.151 W/mK [19,20]. Modified textile materials consist of a polyester yarn and an acrylic coating containing microcapsules. The thermal conductivity for PET and acrylic resin are about 0.15 W/mK and 0.2 W/mK, respectively [21]. Theacrylic coating is a barrier, so it can delay heat access and its emission. It is why the initial temperatures for modified materials shift towards higher melting and lower crystallization temperatures [8]. However, due to the lower amount of MPCM on the textile material, the heat is absorbed faster in relation to the powder, and therefore the temperatures in the peaks maxima of the phase transitions *T_m_* and *T_c_* are achieved faster than for the powder [7,8].

Lower *T_m_* values for modified textile materials compared to MPCM powders were observed by Shin et al. [21] who padded a polyester knitted fabric with polyurethane paste contained *n*-Eicosan microcapsules. Similar results were obtained by Sanchez et al. [10] by coating the cotton woven fabric with polyacrylic paste with MPCM containing a blend of paraffins C_19_–C_27_ also Kim and Cho [22] for a polyester woven fabric coated with polyacrylic paste with MPCM containing *n*-Octadecane. In turn, Chung and Cho [23] studied polyamide fabric, which was coated with polyurethane paste with MPCM containing *n*-Octadecane. They obtained a higher *T_m_* value and a lower *T_c_* value. It may be related to the thermal conductivity of PA (about 0.25 W/mK), but lower for PU (0.019 W/mK) depending on the porosity [21]. The characteristic of *T_m_* and *T_c_* temperatures, seems to be the result of the total material tested but also the conditions of DSC analysis. The reasons of this can be substantiate in the difference in thermal properties of all components of coated fabrics (PET, polyamide, cotton and acrylic and urethane coating), but also in the differences between the material structure, their density and the content of air spaces [21].

**Figure 2:**
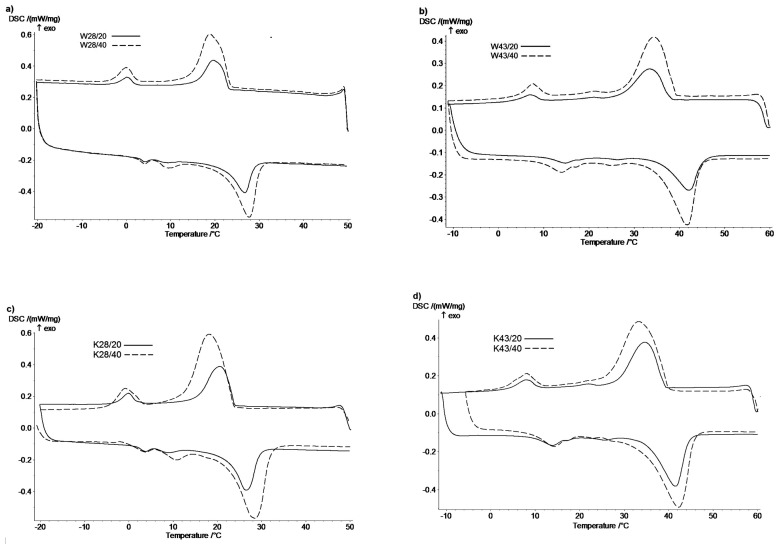
DSC thermograms of woven fabrics with (**a**) MPCM28, (**b**) MPCM43 and knitted fabrics with (**c**) MPCM28, (**d**) MPCM43.

The narrower temperature ranges of phase transitions occur for W and K fabrics modified with a paste containing 20% MPCM (P/20) than the one with 40% (P/40). In the case of W and K fabrics modified with P/40 paste the *T_Onset (S-S_*_)_ temperature of the melting process is lower than for those modified with P/20 paste. Only K43/20 fabric has *T_Onset (S-S_*_)_ temperature slightly lower than K43/40 fabric, which may result from uneven application. The initial temperature of *T_Onset (S-L_*_)_ is higher for both materials modified with paste P/40 than the one with P/20, which is due to the higher content of MPCM in the materials. Higher content of MPCM provides more heat than for smaller quantities. For lower MPCM content, the temperatures at melting peak maxima are lower, and higher in crystallization peak maxima. It is because a smaller amount of MPCM microcapsules allows to reach peaks maxima of phase transitions faster. For initial melting temperatures of K fabrics, lower values of *T_Onset (S-S_*_)_ and higher values of *T_Onset (S-L_*_)_ and higher values of initial crystallization temperatures were also found for comparison with W fabrics. The temperatures at the peak maxima of *T_m_* and *T_c_* are correspondingly higher for K than for W fabrics. The *T_Onset (S-S_*_)_ temperature is lower for modified K fabrics, because of thehigher amount of MPCM on the material than for W fabrics. Although the application of the coating with MPCM on the woven fabric is higher than that on the knitted fabric (Tables 4 and 5). DSC analysis showed the lower melting and crystallization enthalpies (Δ*H_m_*, Δ*H_c_*) by about 45% and 35% for W and K fabrics modified with paste P/20 respectively with MPCM28 and MPCM43 compared to materials with P/40 (Figure 2). Shin et al. [21] padded polyester knitted fabric with paste containing from 5 to 23% of microcapsules with n-Eicosan. They also observed an increase in melting enthalpy (0.91–4.44 J/g) with increasing MPCM content in the paste.

**Figure 3:**
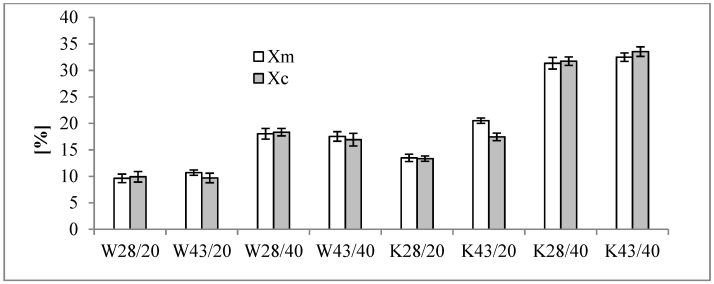
Coefficient of thermal efficiency of melting (*X_m_*) and crystallization (*X_c_*) process fortextile materials modified with MPCM28 and MPCM43.

The values of *X_m_*, *X_c_* were estimated on the basis of melting and crystallization enthalpies values (Δ*H_m_*_,_Δ*H_c_*). The obtained values are shown in Figure 3.

On the basis of the estimated values of *X_m_* and *X_c_*, the coefficient of thermal efficiency of the W28/20 fabric was about 9%, meaning 9% of active MPCM28 is on the W fabric, while on W28/40 it is about 17% (Figure 3). This confirms the results of the microscopic analysis (Figure 1b,c). In turn, the K28/20 and K28/40 K fabrics contain about 17% and 33% of MPCM, respectively, and so the microcapsules have been damaged to a lower extent than on W fabrics (Figure 1e,f). The W43/20 fabric coefficient of thermal efficiency is about 11%, and the W43/40 fabric is about 18% for MPCM43. K fabrics K43/20 and K43/40 contain respectively about 20% and 33% of active MPCM43.

### 2.3. Infrared Thermography

#### 2.3.1. Examination of MPCM Modified Textile Materials during the Heating Process

**Figure 4:**
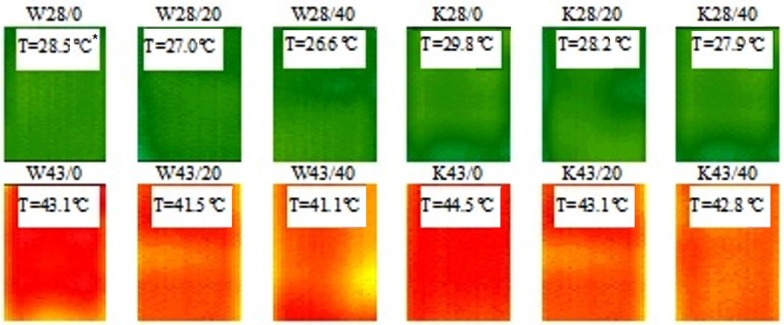
Thermograms of heating process recorded for W28 fabric in 120s and W43 in 270s and for K28 recorded in 110s and K43 in 270s (* average surface temperature of the sample).

**Figure 5:**
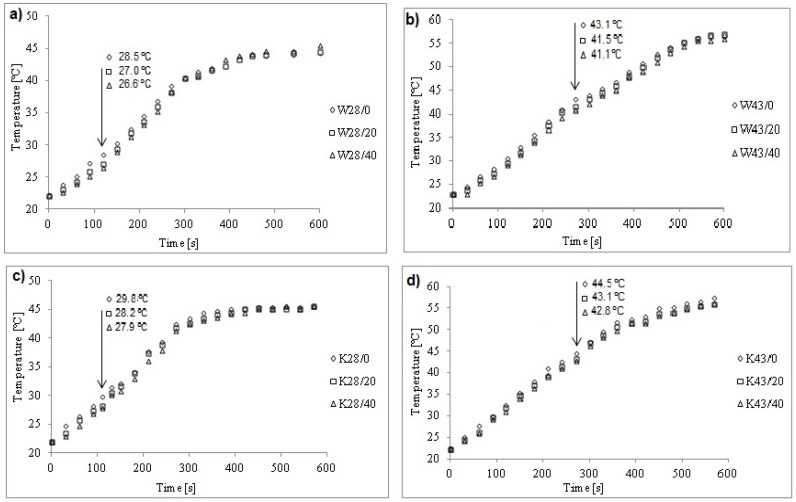
The temperature dependence on heating time for: (**a**) woven fabrics W28/0, W28/20, W28/40; (**b**) wovenfabrics W43/0, W43/20, W43/40; (**c**) knitted fabrics K28/0, K28/20, K28/40; (**d**) knitted fabrics K43/0, K43/20, K43/40.

On the basis of thermograms (Figure 4) registered during the heating process, for modified W fabrics registered in 120s and 270s, respectively and for modified K fabrics recorded at 110s and 270s, respectively, the mean temperature relationships from heating time of W and K fabrics modified with paste without and with MPCM were determined (Figure 5).

**Figure 6:**
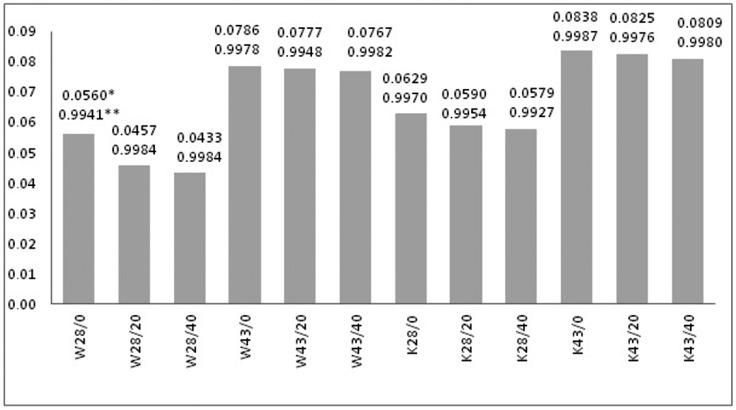
Values of the slope coefficient of the straight line determined from the linear relationship for the heating process (* slope coefficient of the straight line, ** linear coefficient of correlation).

Based on these temperature dependencies, the temperature and time for which there is the maximum temperature difference between W and K fabrics without and with MPCM were determined. The growth rate of the surface temperature was determined from 0s to the time of maximum difference: for W fabrics up to 120s with MPCM28 and 270s with MPCM43, for K fabrics up to 110s with MPCM28 and 270s with MPCM43. It was found that the increase in MPCM content causes the increase of modified materials surface temperature, and its growth rate decreases with the increase of MPCM (Figure 6).

The temperature of materials modified with pastes with 20% rather than 40% of MPCM28 and MPCM43 increased faster. Higher MPCM content results in higher heat accumulation, but slower heating, which is confirmed by DSC results.

During the heating process, materials with MPCM had an average surface temperature lower than materials without MPCM. The temperatures determined on the basis of the heating process in which there is the maximum temperature difference between the materials with MPCM and without MPCM, indicate the melting temperature.

**Figure 7:**
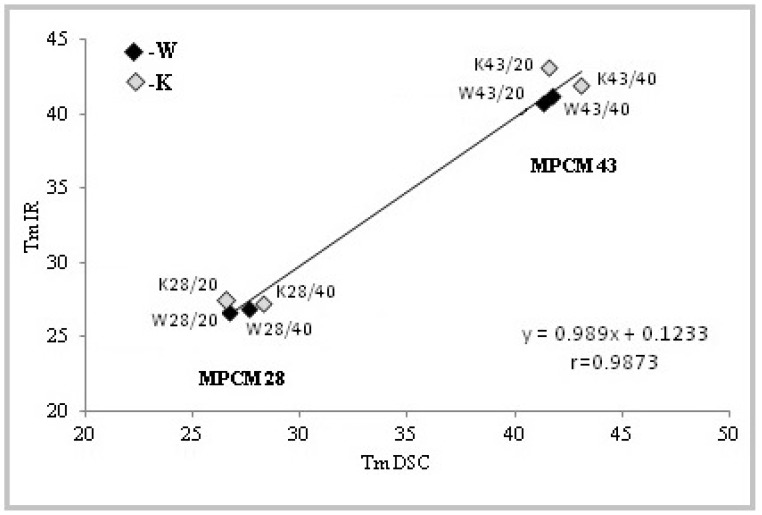
Comparison of MPCM melting temperatures determined by DSC and infrared thermography for woven (W) and knitted fabrics (K) modified with paste P/20 and P/40.

Mean values of melting temperatures (*T_m_IR*) were determined by infrared thermography for W and K fabrics modified with MPCM (Table 2) and compared (Figure 7) with melting temperatures determined with DSC (*T_m_DSC*) (Table 1).

The obtained dependences can be described by a straight line equation with a linear correlation coefficient of 0.9873 (Figure 7).

#### 2.3.2. Examination of MPCM Modified Textile Materials during the Cooling Process

**Figure 8:**
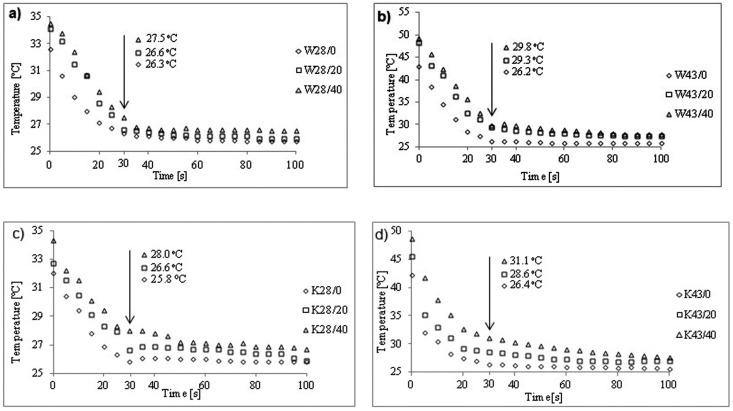
Temperature changes in the surface of materials during cooling process for: (**a**) woven fabrics W28/0, W28/20, W28/40; (**b**) woven fabrics W43/0 W43/20, W43/40, (**c**) knitted fabrics K28/0, K28/20, K28/40; (**d**) knitted fabricsK43/0, K43/20, K43/40.

Based on the thermograms recorded during the cooling process, the mean surface temperature from the cooling time dependence of the MPCM28 and MPCM43 modified materials was determined (Figure 8).

**Figure 9:**
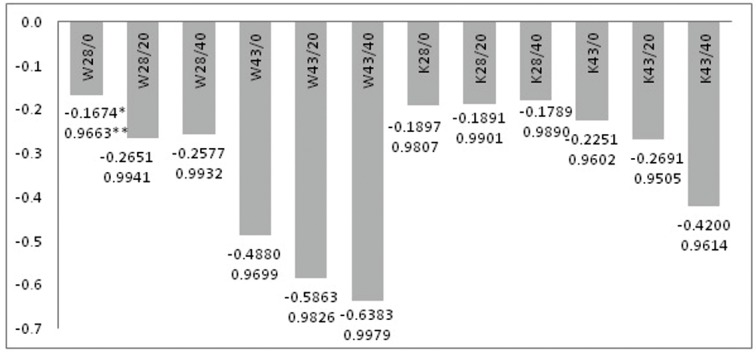
Values of the slope coefficient of the straight line, determined from the linear relationship for the cooling process in the time range 0–30s (* coefficient of slope of the straight line, ** linear correlation coefficient).

From Figure 8, it was found that, the biggest differences are between unmodified W and W43/40 fabric and between unmodified K and K43/40 fabric. This differences amounts respectively 3.6 °C and 2 °C for W fabrics and 4.7 °C and 2 °C for K fabrics, respectively after 30s and 100s of the cooling process. Higher temperatures after 30s for K fabrics than for modified W fabrics in the same way result from both: higher content of MPCM in K fabric determined on the basis of DSC analysis, respectively 17% and 33% in fabrics K43/20 and K43/40 and also 9% and 17% for fabrics W43/20 and W43/40, as well as differences in the rate of temperature decrease. The values of the slope coefficient of the straight line (a) (T = at + b) were determined from the linear relationship in the 0–30s range (Figure 9).

Faster temperature decrease in the 0–30s range was observed for modified W fabrics, which is associated with a more dense W fabric structure than in K fabric. The K fabric has more air-filled spaces (Figure 1d,e) that, due to thermal insulation properties, delay heat transfer. A similar effect was observed by Sanchez-Silva et al. [16] who studied woven fabric being a blend of polyester (82%) and polyurethane (18%) fibers coated with polymer paste containing microcapsules with Rubitherm RT 31. The temperature difference for material with MPCM with respect to material without MPCM was 0.9 °C after 30s of cooling.

## 3. Materials and Methods

### 3.1. Textile Materials and Polymer Pastes

The raw polyester woven (W) fabric (100% PET) with plain weave and polyester knitted (K) fabric (100% PET) with a column weave, with the characteristics given in Tables 4 and 5, were modified. The modification was based on acrylic polymer pastes containing commercial PCM microcapsules (MPCM) (Microtek Laboratories, USA) made of melamine-formaldehyde shell (10–15%) and the paraffin (85–90 wt.%) entrapped inside. The MPCM preparation process was described in previous work [7]. Two types of MPCM were used: *n*-Octadecane (MPCM28) and a mixture of paraffins with the predominance of n-Docosane (MPCM43) with a phase transition temperatures of 28 °C and 43 °C, respectively. The W and K fabrics were coated on one side with the polymer pastes. Pastes contained respectively 0%, 20% and 40% PCM microcapsules and binding agents: Revacryl 123 (Synthomer, Ltd., UK), Helizarin^®^ Fixing Agent TX 4737, Lutexal^®^ Thickener TX 4733 (BST, Germany) (Table 3).

### 3.2. Incorporation Technique

Polymer pastes were incorporated into the textile material structure by coating technique. The coating and heating set MATHIS KTF-350S (Switzerland) was used. The thickness of the air knife gap was 0.05 mm, speed 1.5 m/min, heat temperature 100 °C. The woven and knitted fabrics width was 50 cm.

The characteristics of coated woven and knitted fabric are given in Table 4 and Table 5.

To determine the values of fabrics weight, five samples size 10 cm × 10 cm were tested.

The add-on percentage on the woven and knitted fabrics was determined with Equation (1):

(1) $$Add-on\;\left[\%\right]=\frac{\left(WX\%-Wraw\right)}{WX\%}\;\times \;100\%,$$

WX%- surface weight of modified woven/knitted fabric with paste without (0%) and containing MPCM (20%, 40%); Wraw- surface weight of raw woven/knitted fabric

The thickness of woven and knitted fabrics was tested on GM-70 unit. From the measuring surface (30 cm × 100 cm) ten randomly circle places (20 cm^2^) were chosen. The emphasis of footer was 1 kPa.

### 3.3. Instrumental Techniques

SEM images were taken using a scanning electron microscope VEGA 3 (Tescan, Czech Republic) and magnification of 150× and 1000×. Samples of textile materials were placed on the platform (diameter 12 mm) and fixed with an adhesive carbon disc.

Investigations of thermal properties of MPCM powders and modified textile materials were carried out using the differential scanning calorimeter DSC 204 F1 Phoenix (Netzsch, Germany). Powders and textile materials with a weight 15–27 mg were placed in an aluminum crucible with a pierced lid and scanned twice in the temperature range −20–60 °C with a rate of 5 °C/min under nitrogen (gas flow 20 mL/min). Three samples of each tested materials were studied.

The temperatures of the melting transition(*T_Onset (S-S)_*- initial temperature of solid-solid transition, *T_Onset (S-L)_*- initial temperature of solid-liquid transition, *T_End_*- final temperature) and the crystallization (*T_Onset_*- initial temperature, *T_End_*- final temperature), temperatures in peak maxima of melting and crystallization (*T_m_*, *T_c_*) and enthalpies of melting and crystallization (Δ*H_m_*, Δ*H_c_*) processes were determined.

The temperatures of the melting transition(*T_Onset (S-S)_*- initial temperature of solid-solid transition, *T_Onset (S-L)_*- initial temperature of solid-liquid transition, *T_End_*- final temperature) and the crystallization (*T_Onset_*- initial temperature, *T_End_*- final temperature), temperatures in peak maxima of melting and crystallization (*T_m_*, *T_c_*) and enthalpies of melting and crystallization (Δ*H_m_*,Δ*H_c_*) processes were determined. To identify the efficiency of heat using by MPCM on the modified W and K fabric, the coefficient of thermal efficiency *X%* of the melting (*X_m_%*) and crystallization (*X_c_%*) process was determined with Equation (2).

(2) $$X_{m/c}\%=\frac{\Delta H_{\left(MPCM\;on\;modified\;textile\;materials\right)}}{\Delta H_{\left(MPCM\;powder\right)}}\;\times \;100\%,$$

Δ*H_(MPCM on modified textile materials)_* - enthalpy of melting/crystallizing of MPCM on a modified textile material; Δ*H_(MPCM powder)_* - enthalpy of melting/crystallizing of MPCM powder

Studies using the infrared thermography method was carried out with the MobIR M3 thermal imaging camera (Guide Infrared, China) integrated with the thermal imaging software Guide IrAnalyser, with infrared image analysis, evaluation of the temperature distribution on the surface and determination of the maximum, minimum and average temperature.

Textile materials were put on the coated side on the heating element. The samples were located 0.5 m from the thermal imaging camera. The tests were carried out at temperature of 20 ± 2 °C and RH ± 40%. The heating process was conducted from ambient temperature to 45 ± 1 °C for MPCM28 and to 60 ± 1 °C for MPCM43. During heating, 50 images were recorded for each sample in the time of 0–600s. The cooling process was carried out at 45 ± 1 °C for MPCM28 and 60 ± 1 °C for MPCM43. 50 images were recorded for each sample in the time of 0–100s. Five samples of each tested materials were studied.

## 4. Conclusions

The DSC analysis and infrared thermography technique was used to evaluate thermal properties of woven and knitted fabrics modified with PCM microcapsules with phase transition temperature of 28 °C and 43 °C under the same conditions.

The DSC analysis showed that melting and crystallization temperatures in peak maxima are higher and lower, respectively, for MPCM powders in comparison with MPCM on modified materials. The onset melting temperature of MPCM powders is lower than for MPCM on modified woven and knitted fabrics.

Phase transition processes of P/20-coated materials take place in a narrower temperature range than for P/40. The initial temperature of the solid-solid transition (*T_Onset (S-S)_*) of melting process for woven and knitted fabrics of MPCM28 and MPCM43 is lower for materials coated with P/40 paste. The initial temperature of the solid-liquid transition (*T_Onset (S-L)_*) is lower for materials modified with paste P/20. Due to the lower MPCM content, the temperatures at melting peak maxima are lower and at crystallization peak maxima are higher than for paste P/40. The smaller amount of MPCM allows microcapsules to achieve the maxima peaks of phase transitions faster.

Melting and crystallization enthalpies (Δ*H_m_*,Δ*H_c_*) of woven and knitted fabrics coated with paste P/20 are correspondingly lower by about 45% and 35% relative to P/40 coated materials for both MPCM28 and MPCM43.

The coefficient of thermal efficiency of woven fabrics with P/20 and P/40 for MPCM28 is about 9% and 17% respectively, and for MPCM43 about 11% and 18% respectively, the knitted fabrics modified with pastes P/20 and P/40 contain about 17% and 33% of MPCM28 and about 20% and 33% of MPCM43, thermally active microcapsules. The lower percentage of MPCM on the materials than in the pastes is due to the MPCM damage during the coating process.

The infrared thermography analysis had shown that materials coated with pastes P/20 heat faster thanthose with pastes P/40 with MPCM28 and MPCM43. Higher MPCM content results in higher heat accumulation, but slower heating. This is confirmed by the DSC results. The maximum temperature differences determined on the basis of the heating process between materials with MPCM and without MPCM indicate the melting temperature. There are very good correlations of the melting temperatures values determined by DSC method and infrared thermography.

In the cooling process for modified woven and knitted fabrics the highest temperature decrease was observed in the first 30s. The modified textile materials coated with pastes P/40 for both MPCM28 and MPCM43 were cooled more slowly than for pastes P/20.

During the coating process, the conditions for applying the paste to the structure of the modified material should be adjusted. The combination of two techniques of thermal performance of textiles allows to obtain valuable information.

## Figures and Tables

**Table 1:** Temperatures and enthalpies of phase transitions for MPCM powders and modified woven (W) and knitted (K) fabrics.

Sample	Melting Process					Crystallization Process			
	*T_Onset (S-S)_*^3^ [°C]	*T_Onset (S-L)_*^3^ [°C]	*T_End_* [°C]	*T_m_* [°C]	Δ*H_m_*^2^ [J/g]	*T_Onset_* [°C]	*T_End_* [°C]	*T_c_* [°C]	Δ*H_c_*^2^ [J/g]
MPCM28 [6,7]	−0.6 ± 0.2 ^1^	23.2 ± 0.2	35.2 ± 0.8	28.7 ± 0.5	173.5 ± 1.7	24.5 ± 0.1	−5.2 ± 0.4	17.9 ± 0.5	172.3 ± 2.3
W28/20	2.8 ± 0.2	22.6 ± 0.3	28.9 ± 0.2	26.7 ± 0.2	16.7 ± 1.1	23.3 ± 0.1	−2.2 ± 0.3	19.8 ± 0.2	17.1 ± 1.4
W28/40	2.6 ± 0.2	22.9 ± 0.2	29.9 ± 0.2	27.6 ± 0.1	31.3 ± 0.5	23.5 ± 0.1	−2.9 ± 0.2	18.9 ± 0.1	31.6 ± 0.5
K28/20 [7]	1.6 ± 0.4	22.9 ± 0.1	29.7 ± 0.2	26.6 ± 0.2	23.4 ± 1.0	24.0 ± 0.0	−3.0 ± 0.1	20.5 ± 0.1	23.0 ± 1.1
K28/40 [7]	0.9 ± 0.6	23.0 ± 0.1	31.8 ± 0.1	28.3 ± 0.5	54.4 ± 1.3	23.5 ± 0.3	−4.5 ± 0.5	18.1 ± 0.6	54.7 ± 2.0
MPCM43 [6]	10.7 ± 0.4	36.4 ± 0.2	49.9 ± 0.3	44.0 ± 0.3	155.1 ± 2.0	41.1 ± 0.2	4.0 ± 0.3	33.4 ± 0.2	154.7 ± 2.6
W43/20	11.0 ± 0.2	35.4 ± 0.3	44.4 ± 0.1	41.3 ± 0.2	16.6 ± 1.3	39.1 ± 0.1	4.2 ± 0.2	33.6 ± 0.3	15.0 ± 0.2
W43/40	10.7 ± 0.3	35.8 ± 0.2	44.6 ± 0.3	41.7 ± 0.4	27.2 ± 0.2	39.4 ± 0.3	4.5 ± 0.2	33.8 ± 0.1	26.2 ± 0.2
K43/20	10.4 ± 0.1	36.2 ± 0.2	44.4 ± 0.3	41.6 ± 0.3	31.8 ± 0.9	39.1 ± 0.3	5.1 ± 0.1	33.7 ± 0.5	27.0 ± 0.7
K43/40 [6]	10.9 ± 0.5	36.4 ± 0.2	46.0 ± 1.2	43.1 ± 0.8	50.4 ± 0.5	39.7 ± 0.2	4.5 ± 0.7	34.0 ± 0.8	51.9 ± 0.9

^1^ mean value ± standard deviation. ^2^ Δ*H_m_* and Δ*H_c_* were determined for both solid-solid and solid-liquid transitions. ^3^ *T_Onset (S-S)_* and *T_Onset (S-L)_* are the onset temperatures of respectively solid-solid and solid-liquid transitions.

**Table 2:** Melting temperatures (*T_m_IR*) for woven and knitted fabric determined with infrared thermography.

Sample	W28/20	W28/40	K28/20	K28/40	W43/20	W43/40	K43/20	K43/40
T_m_IR [°C]	26.7 ± 0.34	26.9 ± 0.27	27.4 ± 0.49	27.2 ± 0.52	40.8 ± 0.52	41.2 ± 0.32	43.1 ± 0.64	41.9 ± 0.53

**Table 3:** Characteristics of polymer pastes.

Paste with MPCM	Pastes Characteristic				
	MPCM (wt.%)	Revacryl 123 (wt.%)	Helizarin^®^ Fixing Agent TX 4737 (wt.%)	Lutexal^®^ Thickener TX 4733 (wt.%)	Water (wt.%)
MPCM/0	0	25	2	5	68
MPCM/20	20	20	2	3	55
MPCM/40	40	20	3	1	36

**Table 4:** Characteristics of woven fabrics.

PET Woven Fabric with Plain Weave, Yarn-Staple Fibers		Modified Woven Fabrics				
		Without MPCM	MPCM28		MPCM43	
MPCM content in the coating paste [%]	raw	0%	20%	40%	20%	40%
Sample symbol	W	W 0	W28/20	W28/40	W43/20	W43/40
Weight (W) [g/m^2^]	172.0 ± 0.5 ^1^	200.0 ± 1.7	217.0 ± 1.4	230.0 ± 0.9	220.0 ± 0.8	232.0 ± 1.0
Thickness [mm]	0.43 ± 0.01	0.45 ± 0.00	0.47 ± 0.01	0.51 ± 0.01	0.47 ± 0.01	0.50 ± 0.01
Add-on [%]	-	14	16	25	22	26

^1^ mean value ± standard deviation.

**Table 5:** Characteristics of knitted fabrics.

PET Knitted Fabric with a Column Weave, Yarn- Continuous Fibers		Modified Knitted Fabrics				
		without MPCM	MPCM28		MPCM43	
MPCM content in the coating paste [%]	raw	0%	20%	40%	20%	40%
Sample symbol	K	K 0	K28/20	K28/40	K43/20	K43/40
Weight (W) [g/m^2^]	73.0 ± 0.7 ^1^	95.0 ± 0.9	108.0 ± 1.1	125.0 ± 0.7	106.0 ± 0.6	131.0 ± 1.7
Thickness [mm]	0.28 ± 0.01	0.26 ± 0.01	0.30 ± 0.02	0.39 ± 0.02	0.30 ± 0.01	0.34 ± 0.01
Add-on [%]	-	23	32	42	31	44

^1^ mean value ± standard deviation.
